# An Exploratory Data Analysis on the Influence of Role Rotation in a Small-Sided Game on Young Soccer Players

**DOI:** 10.3390/ijerph18136773

**Published:** 2021-06-24

**Authors:** Moisés Falces-Prieto, Francisco Tomás González-Fernández, Jaime Matas-Bustos, Pedro Jesús Ruiz-Montero, Jesús Rodicio-Palma, Manuel Torres-Pacheco, Filipe Manuel Clemente

**Affiliations:** 1 Research Center High Performance Soccer, Fundación Marcet, 08035 Barcelona, Spain; mfalpri@gmail.com (M.F.-P.); jesus.rodicio@gmail.com (J.R.-P.); mtorpac@gmail.com (M.T.-P.); 2 Department of Physical Activity and Sport Sciences, Pontifical University of Comillas, 07013 Palma, Spain; francis.gonzalez.fernandez@gmail.com; 3 SER Research Group, Pontifical University of Comillas, 07013 Palma, Spain; 4 Department of Architecture and Computer Technology, University of Granada, 18010 Granada, Spain; jmatasbustos@gmail.com; 5 Department of Physical Education and Sport, Faculty of Education and Sport Sciences, Campus of Melilla, University of Granada, 52071 Melilla, Spain; pedrorumo@ugr.es; 6 Escola Superior Desporto e Lazer, Instituto Politécnico de Viana do Castelo, Rua Escola Industrial e Comercial de Nun’Álvares, 4900-347 Viana do Castelo, Portugal; 7 Instituto de Telecomunicações, Delegação da Covilhã, 1049-001 Lisboa, Portugal

**Keywords:** football, drill-based games, performance, task constraints, external load, youth soccer

## Abstract

The aim of the present study was to analyze the behavior of players in a standard small-sided game (SSG) according to the role played (offensive (OF), defensive (DF), and wildcard (W)) and its relationship with physical demands (PHYD), technical performance (TP), and internal load (RPE). A total of 24 young highly trained male soccer players (under 16: *n* = 12; under 19: *n* = 12) participated. During the SSG, the players alternated the three roles (OF, DF, and W). The duration of each repetition was 4 min with a passive rest of 3 min between them. Furthermore, it emphasized the high demand in all defensive parameters. In addition, DF roles showed higher values in PHYD and RPE, followed by the OF roles, and finally by the W roles. A complementary, positive moderate correlation was found between PHYD and RPE in the U16 dataset (r = 0.45, *p* < 0.006). Very large positive correlations were also found between PHYD and RPE in the U19 and merged dataset (r = 0.78, *p* < 0.001 and r = 0.46, *p* < 0.63, respectively). This information could be useful for coaches in order to structure the roles in SSGs and control training load.

## 1. Introduction

Small-sided games (SSGs) are one of the most commonly used tasks during soccer training at all ages and competitive levels [1]. These drill-based games can be characterized as being adjusted formats regarding the formal game, in which specific task constraints change the player’s responses in accordance with the objective of the exercise [2]. Among different task constraints used in the design of SSGs, the adjustment of the number of players involved (format) is one of the most commonly used and researched [3,4]. By employing a reduced number of players, the coaches’ principal aim is to increase the ratio of players’ participation time with the ball while maintaining some technical and tactical demands that often are implied in matches [5,6]. The greater ratio of players´ time with the ball obtained during SSGs, in comparison with habitual competition, has two main benefits during the process of soccer conditioning. Firstly, there is greater physiological stress per minute of the game [7]. In fact, SSGs have been proposed as optimal training drills to develop specifically the soccer player’s endurance capacity [8]. Secondly, SSGs are an efficient way to promote technical and tactical behaviors in the players [9,10]. Although it is a training strategy widely used by coaches and fitness coaches, there is still a gap about how it affects the process of adaptation to space and the variation of roles within the same task, about physical demands, technical performance, and load control.

The effect of SSGs depends on the presence of a series of task constraints, including field size [11], different surfaces [12], the orientation of the game space [13] coach encouragement [14], number of players per team [15], the presence/absence of the head coach [1], the presence/absence of the wildcards [16,17] or of goalkeepers [18], and more recently studied, the order in which the SSGs are presented during the session as a load conditioning element [19]. In this respect, by manipulating these variables, it is possible to modify the demands of the game and the implication of muscular and mental fatigue [20]. However, there is no scientific literature on the effect of role change within the same task and the development of the same task in different categories.

Different monitoring instruments have been used to properly quantify and qualify the impact of task constraints on the players [21]. Technological advances have helped in the analysis and quantification of physical demands owing to the incorporation of global positioning systems (GPSs) [22]. GPSs have been used to describe the physical demands (PHYDs) of the soccer player during training, as well as friendly and official matches [23]. Accordingly, at the elite level of sport, the use of GPS is becoming more commonplace, with the main aim of reporting the daily demands imposed on both the individual and the team [24]. Using microelectromechanical systems, it was possible to observe that larger pitch sizes (mainly longitudinal) are favorable for increasing high-intensity running and sprints while reducing the pitch size increases the density of accelerations [25].

While the external load represents the physical demands imposed by the drill, the internal load represents the physiological responses to the external load [26]. One of the most common measures related to internal load is the rate of perceived exertion (RPE) [1,27]. RPE has previously been defined as a valid instrument to quantify the intensity levels or internal load achieved in a task [28,29]. Furthermore, RPE has commonly been used optimally to control the load that is generated in the players during SSG [30]. In addition, RPE increases in smaller formats of play, regarding relative area per player and with the coach’s encouragement [1,31].

Another important concept in this research is technical performance (TP). In this regard, SSGs play an important role in the acquisition and refinement of technical and tactical aspects [1]. Naturally, because the dynamics of the sport are maintained during SSGs, games influence the emergence of different adaptive behaviors during the match, thus leading to low-to-moderate reproducibility of the technical actions performed during SSGs [6,32]. Nevertheless, it is expected that changes in different task conditions may have different effects on players’ technical performance [6]. As an example, reducing the format of play and the relative area per player meaningfully increases the number of individual technical actions while increasing the pitch size increases ball possession [3].

Despite a substantial body of knowledge related to SSGs and their impact based on different task constraints [3], it is still necessary to understand the impact of the different roles in the same task since this is not a regularly analyzed topic in the research on SSGs. This may help to understand the eventual heterogeneity of the training stimulus based on different roles and provide useful information for coaches to adjust the practice. Therefore, the aim of the present study was to analyze the behavior of players in a standard SSG according to the role played and its relationship with physical demands, technical performance, and internal load. The hypothesis was raised that the defensive role is the one with the highest perception of effort and physical demands and the one with the least wild cards. On the other hand, given that muscular and mental fatigue affects the players’ opportunities for action, the effectiveness of the passes can be expected to increase as the task progresses, since the players will adapt to the playing space.

## 2. Materials and Methods

### 2.1. Study Design

This study followed a nonrandomized cross-sectional design to determine the behavior of the players in a standard positional game according to the role played. These players practiced 5 times per week, 90 min per session, with an official competition every weekend. The training sessions were based on technical and tactical content development (70% of training time), technical skill improvement (10% of training time), and general improvements in physical condition (20% of training time). Generally, training sessions comprised a warm-up, main part, and cool down. Data were collected during the month of November in the 2019–2020 season. The research was carried out on day −4 (Wednesday) of a normal training week. Subjects were randomly assigned to 3 groups: offensive (OF), defensive (DF), and wildcard (W). The positional game format was (4OF vs. 4DF + 4 W) without the goalkeeper. Both categories (U16 and U19) performed the same SSG format. To determine the effects of the SSGs, the following variables were selected: (a) physical demands with GPS, (b) technical performance with a video camera, and (c) internal load using RPE.

### 2.2. Participants

A total of 24 healthy highly trained young male soccer players participated in the study. The players were grouped into two categories: under 16 [(*n*= 12); age: 14.41 ± 0.51 years; height: 170.87 ± 5.14 cm; weight 59.18 ± 7.43 kg; CMJ: 34.59 ± 3.54 cm; VO_2_ max: 50.86 ± 1.41 mL·kg^−1^·min^−1^)]; and under 19 [(*n*= 12); age: 17.33 ± 0.65 years; height: 177.08 ± 7.73 cm; weight 64.55 ± 6.10 kg; CMJ: 38.03 ± 4.10 cm; VO_2_ max: 52.35 ± 2.07 mL·kg^−1^·min^−1^)]. Inclusion criteria for participants in this study were, on the one hand, that they reported normal or corrected to normal vision, no partial/chronic injury, and no history of a neuropsychological impairment that could affect the results of the experiment, and moreover, an average experience in licensed soccer of ~5 years. All the parents and participants were informed about the purpose of the study and signed the consent form detailing the possible benefits and risks. The participants were fully debriefed about the purpose of the study at the end of the experiments. The study was conducted in accordance with the ethical standards established by the Helsinki Declaration of 2017.

### 2.3. Physical Demands (PHYD)

The players’ positioning was recorded using GPS devices operating at a sampling frequency of 10 Hz and integrated with 400 Hz Tri-Axial accelerometers and Gyroscope (Playertek^®^, Catapult Innovations, Dundalk, Ireland) [33]. Playertek appears to be more precise in speed estimation across different weather conditions [34]. However, there are currently no studies carried out on these units because they are relatively new additions to the GPS market. Players were required to wear a GPS unit fitted on the upper back of each player using a neoprene harness. All the units were activated more than 20 min before training, allowing the acquisition of satellite signals [35]. To avoid interunit error, each player used the same GPS device throughout the study period. The recorded variables were the total distance covered (TDC), distance covered per minute (DPM, m·min^–1^), distance covered at high velocity (DHV, >18 km·h^−1^, m·min^–1^), total number of accelerations (TNA), and decelerations (TND).

### 2.4. Technical Performance (TP)

A recorder system with a video camera (Sony, model HDR-CX190 High Definition Handycam 5.3 MP Camcorder), placed at a fixed tripod height and lateral to the field was used for visualization and subsequent quantification of technical actions. The quantified technical actions were the successful passes (SP) made when playing the offensive (OF) and wildcard (W) roles and the interceptions (INT) made when playing the defensive role (DF). This quantification was performed using Match Vision Studio Premium [36,37] and Microsoft Windows^®^ Excel program. The main observer has more than 8 years of practical experience as a fitness coach and soccer researcher. The reliability of the recorded data was estimated by intraobserver reliability, which consists of the observer–researcher analyzing the same data again on another occasion. In the case of this study, the main observer–researcher visualized the technical actions two days later. The results obtained in the first and second evaluations were then compared. Furthermore, the reliability of the data recorded by the observer–researcher was calculated using Cohen’s kappa coefficient of agreement, where values of 1.00 were obtained; therefore, according to the evaluation scale proposed by Landis and Koch [38], in all the calculated Cohen kappa coefficients, an almost perfect agreement strength was achieved.

All technical actions were recorded individually during the analysis of the positional game [1]. Technical actions were defined and organized as (a) SP: A pass performed with the foot or head that is received successfully by a teammate and (b) INT: When an opposing player cuts the path of the ball between two players of the same team, using different body parts for contact (foot, heel, thigh, knee, chest, or head).

### 2.5. Internal Load: RPE

The CR-10 Borg scale [29] was obtained immediately after each set of SSGs. All the players were familiar with this tool, using it daily both in training and in competition. During the data collection, they chose a score individually from 0 to 10 to the question “How intense was the exercise?” They were allowed to answer in 0.5 units. In this case, it was the fitness coach who was responsible for recording the data. For this purpose, he asked each player in private (without the presence of other teammates and without them being able to see the values set by the rest of the teammates), how demanding the game had been for him. All data were collected in paper format and were subsequently recorded in a spreadsheet in the Microsoft Windows^®^ Excel program (Redmond, WA, USA).

### 2.6. Small-Sided Game (SSG)

The study was conducted in the month of November of the 2019/2020 season, 11 weeks after the start of the season. The research was carried out on day −4 (Wednesday) of a normal training week for both groups. Both groups (U16 and U19) performed the task simultaneously. All training sessions were held at the same time of day (16:00–17:00 p.m.). The evaluations were carried out at the same time of day and under similar meteorological conditions (~22 °C and ~60% humidity). Subjects were randomly assigned to three groups, which were maintained throughout the task. The three teams were created by the head coach to ensure no technical/tactical differences existed between them. The format of the SSG was of the positional game (PG). Players were familiarized previously with the SSG used during the study. The PG was (4OF vs. 4DF + 4 W) without the goalkeeper. The aim for the OF and W roles was continuity of play and the creation of passing lanes. For its part, the DF role aimed to intercept the greatest number of passes and prevent the progression of the game.

**Figure 1:**
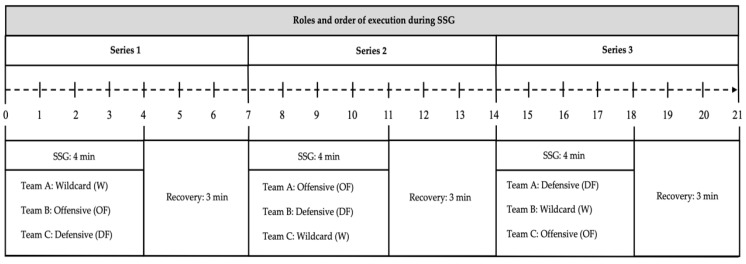
Roles and order of execution during the SSG.

The pitch size was 20 × 20 m, such that the relative area per player was 50 m^2^, excluding the W. The duration of each repetition was 4 min, with a passive rest of 3 min between them. The number of ball touches was unlimited and when the ball came out of the playing area or was intercepted, the fitness coach quickly provided another ball ensuring maximum effective playing time [1]. Before the PG, participants performed a 10 min standardized warm-up that included jogging, dynamic stretching, and joint mobility exercises. The start and end signals were whistled by the fitness coach. The roles and order of execution are shown in Figure 1.

### 2.7. Design, Data Reduction, and Statistical Analysis

An exploratory data analysis (EDA) was used on the dataset mentioned. In statistics, exploratory data analysis refers to the critical process of performing initial investigations on data to discover patterns, spot anomalies, test hypotheses, and check assumptions with the help of summary statistics and graphical representations [39,40]. It also encourages statisticians to explore the data and possibly formulate hypotheses that could lead to new data collection and experiments.

To achieve that goal, we used toolstack SciPy [41], a free software ecosystem used by data and engineering scientists to carry out their scientific studies and analyses on datasets [41,42], and implemented them in the Python programming language. Specifically, we used the environment for scientific computing IPhyton [43], together with the Pandas [44] and Numpy [41,45] libraries, for statistical computing to perform data processing. In addition, we used the specialized library Matplotlib to design and generate the graphics for this analysis [46]. All variables were normalized to domain [0, 1], using the following formula, known as min–max normalization or feature scaling:

(1) $$x\prime =\frac{x-\min\;(x)}{\max\;(x)-\min\;(x)}$$

During the normalization process, maximum and minimum of observations in both categories (controlled by the main researcher) were used for all variables; therefore, the scaling of each observation with maximum and minimum values corresponded to each category (except RPE and SP/INT variables). On the one hand, in RPE, a maximum value of 10 and a minimum of 0 were used with the aim of conforming to the representation of the Borg scales independent of the category. On the other hand, for SP/INT with the aim of faithfully representing what success in each type of role entails while being fair, the processing was carried out so that each observation was normalized using the maximum and minimum depending on the category and roles to which it belonged. For example, due to the type of game and configuration of players, it is much more complicated to achieve success in the defensive role than in the others.

Subsequently, the PHYD variable was constructed grouping normalized TDC, DHV, DPM, TNA, and TND variables in order to represent as faithfully as possible and under a single vision, the physical demands of the subjects studied. For this reason, a linear model was used and is represented by the following equation:

(2) y=w · x+ξ

The equation is laid out in such a way that

(3) $$\mathrm{PHYD}=\overset{n}{\underset{i=1}{\sum }}(w_{i}\;\cdot \;x_{i})+\xi$$

Residual value being *ξ* = 0, our vector of variables *x*, of size *n* = 5, including the following variables:

(4) $$x=\left\{x_{1}=\mathrm{TDC}\;,\;x_{2}=\mathrm{DHV},\;x_{3}=\mathrm{DPM},\;x_{4}=\mathrm{TNA},\;x_{5}=\mathrm{TND}\right\}$$

In this respect, as noted earlier, in the present study it was decided to give equal importance to all variables of vector *x* so that the vector of weights, *w*, was shaped as follows:

(5) $$w=\left\{w_{1}=0.2\;,\;w_{2}=0.2,\;w_{3}=0.2,\;w_{4}=0.2,\;w_{5}=0.2\right\}$$

After performing all previous processing, all variables were worked by grouping them into three datasets: the dataset of the U16 category, dataset of the U19 category, and the “merged” dataset, which includes both categories.

Finally, a Pearson correlation coefficient *r* was used to examine the relationship between values of physical demands (PHYD) and internal load (RPE). To interpret the magnitude of these correlations we adopted the following criteria: *r* ≤ 0.1, trivial; 0.1 < *r* ≤ 0.3, small; 0.3 < *r* ≤ 0.5, moderate; 0.5 < *r* ≤ 0.7, large; 0.7 < *r* ≤ 0.9, very large; and *r* > 0.9, almost perfect.

## 3. Results

The following figures present a set of 18 annotated heatmaps for the study of physical demands, technical performance, and internal load. In all the figures, the dark colors show higher rates for each variable. On the contrary, the light color indicates less prevalence.

### 3.1. Physical Demands (PHYD)

**Figure 2:**
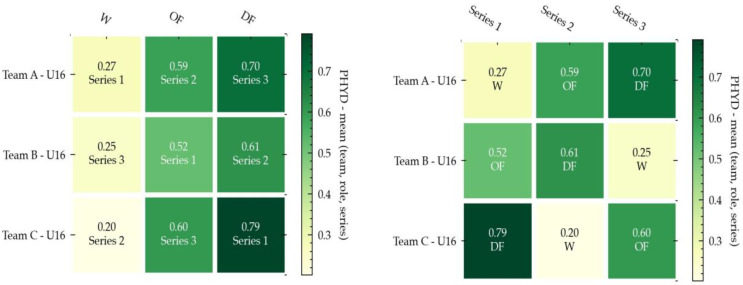

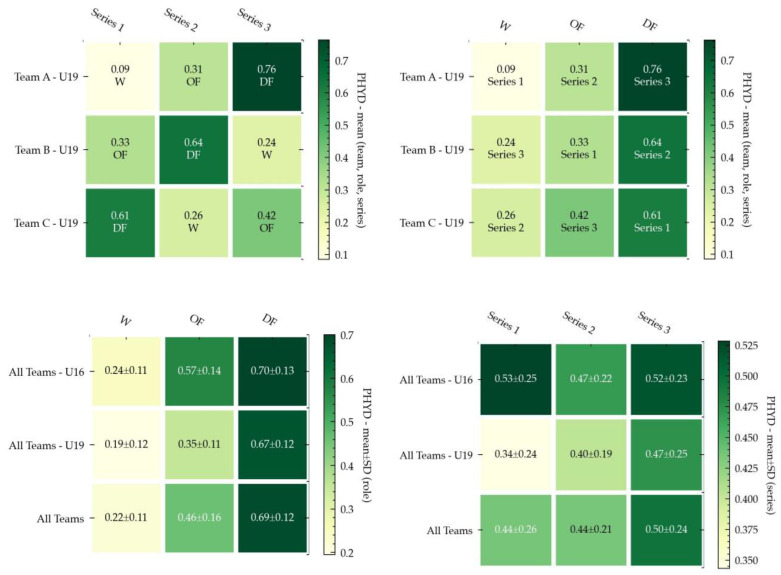
Annotated Heatmaps for the study of the PHYD variable (U16, U19, and datasets merged). Note: The following figure presents six heatmaps for the study of physical demands in the three datasets studied, in which first row shows U16 results, the second shows U19 results, and the third shows merged dataset results. The three heatmaps in the left column focus on the comparison among roles (OF, DF, and W), while heatmaps in the right column focus on the comparison among series (Series 1, Series 2, and Series 3). Dark colors show a higher PHYD rate, whereas the light color indicates less prevalence.

PHYD are represented in the set of six heatmaps in Figure 2, which show the comparison of different teams (Team A, Team B, and Team C), different roles (OF, DF, and W), and lastly, the different series (Series 1, Series 2 and Series 3). The data analysis in Figure 2 shows that the DF role was the one with the highest physical demands, followed by the OF role, and finally the W role. PHYD implied significantly more demands in the third series.

### 3.2. Technical Performance (TP)

**Figure 3:**
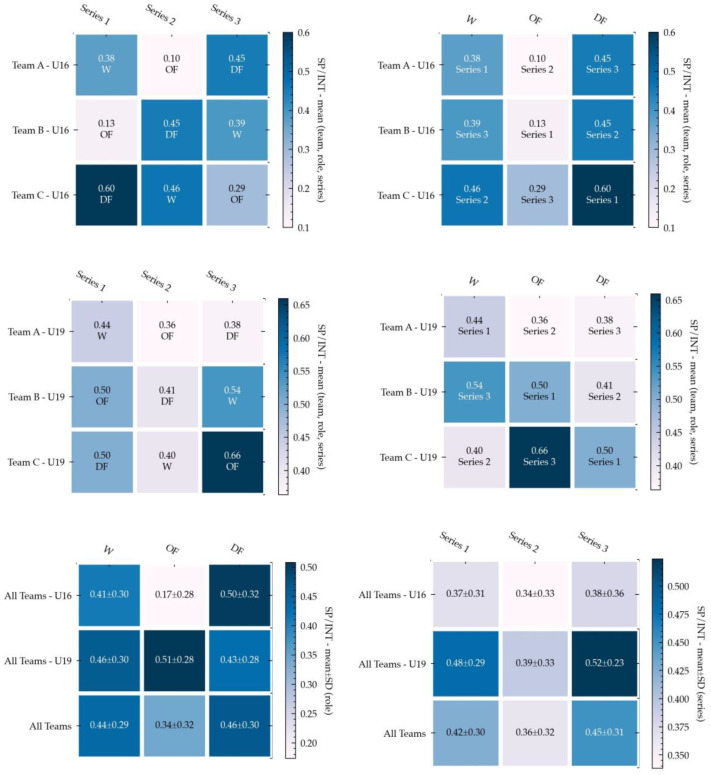
Annotated Heatmaps for the study of the SP/INT variable (U16, U19, and datasets merged). The following figure presents six heatmaps for the study of technical performance in the three datasets studied, in which the first row shows U16 results, the second shows U19 results, and the third shows merged dataset results. The three heatmaps in the left column focus on the comparison among roles (OF, DF, and W), while heatmaps in the right column focus on the comparison between series (Series 1, Series 2, and Series 3). Dark colors show a higher PHYD rate, whereas the light color indicates less prevalence.

TP is represented in another set of six heatmaps (Figure 3) showing the comparison of different teams (Team A, Team B, and Team C), with different roles (OF, DF, and W) and different series (Series 1, Series 2 and Series 3). The data analysis in Figure 3 shows that the results of TP variables clearly depend on category. In the U16 group, teams with OF roles achieved less success or accuracy during the game, while teams with the DF role were very accurate. In the U19 group, results were more balanced regarding accuracy. Teams in OF or W were less reliable, inaccurate, and variable.

### 3.3. Internal Load: RPE

**Figure 4:**
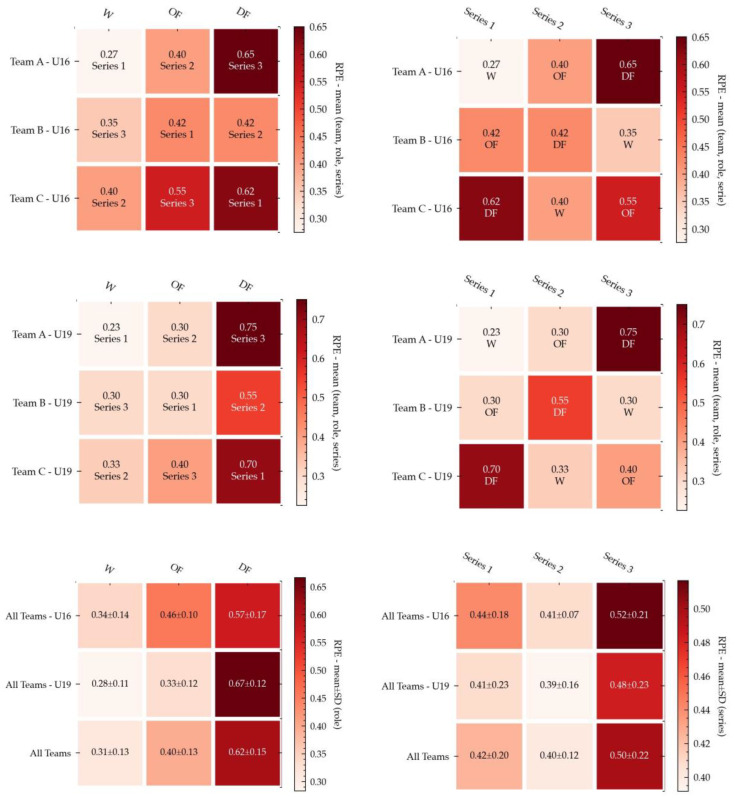
Annotated heatmaps for the study of the RPE variable (U16, U19, and datasets merged). The following figure presents six heatmaps for the study of internal load in the three datasets, in which the first row shows U16 results, the second shows U19 results, and the third shows merged dataset results. The three heatmaps in the left column focus on the comparison among roles (OF, DF, and W), while heatmaps in the right column focus on the comparison between series (Series 1, Series 2, and Series 3). Dark colors show a higher PHYD rate, whereas the light color indicates less prevalence.

Finally, RPE is represented in the last six heatmaps shown in Figure 4. First, we can observe the comparison of different teams (Team A, Team B, and Team C), with different roles (OF, DF, and W) and different series (Series 1, Series 2, and Series 3). The data analysis in Figure 4 shows higher values in DF roles than in OF roles or W roles. The data revealed that this situation appears in all categories in the same way. RPE data coincided with PHYD values in reference to the roles and series.

### 3.4. Correlations Analysis (PHYD vs. RPE)

**Figure 5:**
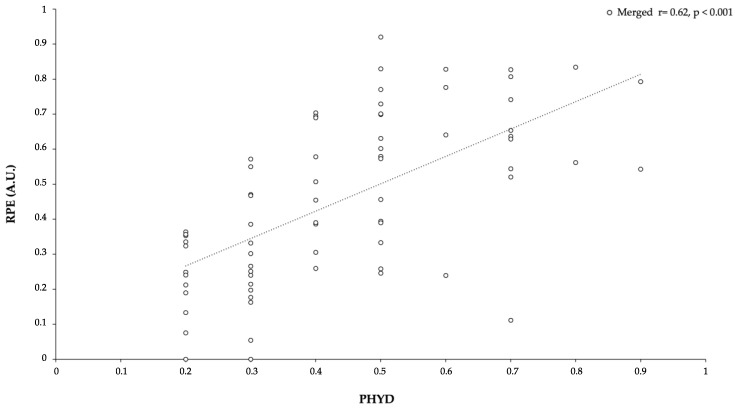
Relationship between PHYD performance and RPE (datasets merged).

**Figure 6:**
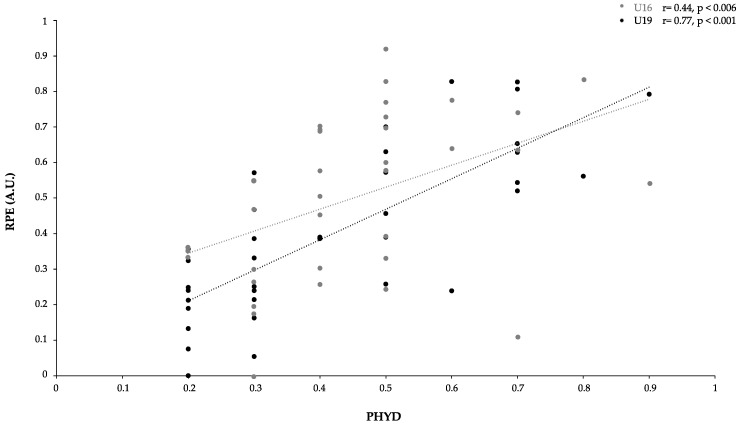
Relationship between PHYD performance and RPE (U16 and U19 categories).

At this point, we wondered whether the relative effect on PHYD performance with respect to RPE was significantly different between categories. We selected the data on RPE from each category (U16, U19, and datasets merged). A Pearson correlation coefficient *r* was used to determine the relationship between PHYD and RPE. Significant correlations were found, as we observed a large correlation between PHYD and RPE (r = 0.63, *p* < 0.001) in the merged dataset (see Figure 5 for more information), a very large one (r = 0.78, *p* < 0.001) in the U19 dataset, and a moderate one (r = 0.45, *p* < 0.006) in the U16 dataset (see Figure 6 for more information).

## 4. Discussion

The aim of the present study was to examine, in highly trained elite youth soccer players, the behavior of players in a standard SSG according to the role played and its relationship with the PHYD, TP, and RPE. The main results of the experiment showed that PHYD values depend on the role played (OF, DF, and W) in the SSG and the sequence of execution. These results have no association with TP in either category. However, internal load reflected in RPE values showed, in the same way as PHYD values, that they depend on the role played. To summarize, DF roles showed higher values in PHYD and RPE, followed by OF roles, and finally by W roles. A novel aspect of this research was the analysis of the variables (EDA). On the one hand, analysis and methodology based on exploratory data analysis was an innovative contribution to the sports science literature and specifically, the physical training of soccer players. On the other hand, this analysis can help to generate specific methodologies for professional teams. Our analysis suggests a more effective decision-making process and enables individualized, analytic, and optimized monitoring.

Previous studies have shown that competition demands oscillate significantly according to the different roles in SSGs [7,47]. In fact, it is well known that over the course of time in SSG accuracy, mistakes and fatigue appear in the session [20,48]. However, all previous studies have investigated competitive demands in terms of external load (e.g., total distance or high-intensity actions), internal load (i.e., RPE), or technical performance (e.g., successful passes or interception), and to the best of our knowledge, there are no studies that analyze the exact moment of player participation in SSGs during soccer training. This aspect must be highlighted because, in a reduced group of soccer players in routine training, the last player has to wait longer than anyone to perform the specific exercise and could be exhausted. Therefore, the role of the soccer player in the SSG is negatively affected by the moment of participation.

The results of the present study suggest, in terms of PHYD values, that the DF role was the one with the highest PHYD, followed by the OF role, and finally the W role. These findings are consistent with the results of a study by Torrents et al. [49] in which the defensive players had to play 1 vs.1 with opponents and increase the technical actions through defensive patterns, the number of passes, or driving between players, with possession of the ball. Regarding the temporal series, Figure 1 seemed to suggest balanced physical work in all series, considering the merged dataset. However, data showed the third series implied significantly more demands in the PHYD variable. With respect to category (U16 and U19), in the U19 group, the PHYD variable was constantly increasing from the first series to the third series. Nevertheless, in the U16 group, PHYD was maximum in the first series, was minimum in the second series, and in the third series, was similar to the first but slightly lower. In other words, the U16 team appeared to lack the mastery of task timing possessed by the U19 team [49]. Moreover, those authors affirm that PHYD, in its broadest sense, could be negatively influenced according to the playing position, specifically in U16 soccer players. Our data suggest that there is no relationship among all players, mainly because the players are adapted to the external demands of SSGs.

The results on the TP variables clearly depend on the category. In the U16 group, teams playing OF roles achieved less success or accuracy during the game, while teams playing DF roles were very accurate. In the U19 group, results were more balanced regarding accuracy. These results are in line with previous research in SSGs that suggests the beneficial effects of experience in performing technical actions more accurately. A comparative study between age groups (under 15 vs. under 17) revealed that older players presented more individual actions than younger ones [50], and both attacking and defensive tactical behaviors tended to be more accurate as players become older [51]. In fact, older players tend to play wider than younger ones, although with large variability that seems to be functional for attacking [52]. At a defensive level, older players tend to perform more concentration behaviors and act together, thus improving accuracy in defensive behavior [53]. Thus, it should be expected that experience and quality of decision making meaningfully contribute to explaining the superiority of attacking and defensive actions in older players [54].

Regarding the effects of roles on technical performance, it was possible to observe that OF and W were less reliable, inaccurate, and variable. Although there is little research about the effects of playing positions in technical performance, a study conducted on under-14 players during a 3 vs. 3 format [54] revealed no differences between playing positions in technical execution. However, this can be caused by the smaller number of players involved, thus increasing their individual participation and involving a less structured context than larger formats of play [54]. Interestingly, in the current research, playing OF and W roles increased the variability, possibly explained by the need to change action execution to solve the issues for overcoming the opponent’s team. The higher variability while performing attacking actions was also observed in a previous study conducted on young soccer players during SSGs [55], supporting this idea. The results of RPE values showed higher values in DF roles than in OF or W roles. The data revealed that this situation appeared in all categories in the same way. Therefore, data suggest that high external PHYD values could present an internal load reflected in RPE similar to previous studies in which physiological and physical variables in SSGs with amateur players were measured as moderate- to high-intensity exercises [56]. Similarly, moderate correlations were established between PHYD and RPE (r = 0.45), and very large ones between PHYD and RPE for the U19 group and merged datasets (r = 0.78 and 0.63), respectively. Thus, the relationship between PHYD and RPE may be due to the greater familiarity with RPE scales in more experienced soccer players (U19) than in soccer players with two or three years less experience.

In addition, SSGs in 2 vs. 2, 4 vs. 4, and 6 vs. 6 formats in young soccer players produce better-perceived exertion and good reproducibility regarding time and duration in total distance and metabolic power. Thus, it would be beneficial to analyze soccer players’ RPE to quantify PHYD and RPE values in order to have an accurate knowledge of the players´ real demands during training sessions and specifically, in roles performed in SSG [5]. Considering temporal series, RPE values change with respect to PHYD values, as shown in the merged dataset. For this reason, they seemed to suggest balanced physical work in the first and second series, but the third series implied a higher internal load for soccer players. Taking category into account, we observed that in the U19 group, PHYD values were different due to RPE increasing from the first series to the third series, being lower in the second series than in the other two series. Lastly, the results of the U16 soccer players were not in normal order—the third series was maximal, followed by the first series, and finally, series 2 with lower values. These results are not consistent with the PHYD measures. However, in our investigation, higher values of RPE were reported in DF roles than other roles, concurring with previous studies because efforts are assimilated with more difficulty in younger (U16) than in older soccer players (U19).

The present study had some limitations. On the one hand, we performed the study without a control group. On the other hand, we used one group of each category (U16 and U19). In this sense, the ideal scenario would have been to put into practice with more groups. In fact, future studies should increase the overall sample for making better generalizations. Lastly, the study was performed on the same day, while this represents a real training situation that could be realized in three different days, with the same group in counterbalanced order to reach a good approach to the problem. Notwithstanding the above, we might consider the design performed as a good approximation to understand new variables that affect the performance of SSG.

Despite the study’s limitations, to the best of our knowledge, there are no previous studies investigating the relationship of the soccer player’s role and physical demands, technical performance, and internal load with this type of analysis.

## 5. Conclusions

The findings of this study demonstrate that PHYD values depend on the role played (OF, DF, and W) in SSGs and on the sequence of execution. These results have no association with TP in either category, which we suggest may be due to differences in TP according to the category. However, internal load reflected in RPE values showed that it depends on the role played. To summarize, DF roles showed higher values in PHYD and RPE, followed by OF roles, and finally, W roles. This information could be useful for coaches in order to structure the roles in SSGs and control training load. Nonetheless, it is also suggested that internal load monitoring cannot be confidently used in isolation as a tool to detect differences in competition demands in young soccer players. Thus, the use of both internal and external load is suggested to manage the training and match load and to prescribe the training sessions appropriately. EDA could help to generate specific methodologies for professional teams. Thus, our analysis suggests more effective decision making to enable individualized, analytic, and optimized monitoring. In fact, provided that this is requested, we will certainly return to this analysis once we have had more experience to change factorial weight. To summarize, the present empirical study demonstrates that the use of SSGs in both U16 and U19 categories is influenced by the relationship of the soccer player’s role and physical demands, technical performance, and internal load. It also contributes to analyzing the importance of RPE and PHYD according to different roles/positions in SSGs, with special emphasis on DF players.
